# Salivary Biomarkers as Prognostic Tools in Oral Squamous Cell Carcinoma: A Systematic Review of Survival and Progression Outcomes

**DOI:** 10.3390/dj13100479

**Published:** 2025-10-17

**Authors:** Matteo Pellegrini, Maurizio Pascadopoli, Mario Romolo Faretta, Alessandro Nobili, Carlos Pérez-Albacete Martínez, Francesco Spadari, Andrea Scribante

**Affiliations:** 1 Section of Dentistry, Department of Clinical, Surgical, Diagnostic and Pediatric Sciences, University of Pavia, 27100 Pavia, Italy; 2 Department of Biomedical, Surgical and Dental Sciences, University of Milan, 20122 Milan, Italy; 3 Maxillo-Facial Surgery and Dental Unit, Fondazione IRCCS Cà Granda Ospedale Maggiore Policlinico, 20122 Milan, Italy; 4 Department of Experimental Oncology, European Institute of Oncology (IEO) IRCCS, 20139 Milan, Italy; 5 Department of Health Policy, Istituto di Ricerche Farmacologiche Mario Negri IRCCS, 20156 Milan, Italy; 6 Tissue Regeneration and Repair Group, Biomaterials and Tissue Engineering, Faculty of Health Sciences, UCAM-Universidad Catòlica San Antonio de Murcia, Guadalupe, 30107 Murcia, Spain

**Keywords:** liquid biopsy, multi-omics, oral squamous cell carcinoma, prognostic indicators, salivary biomarkers

## Abstract

**Background/Objectives**: Oral squamous cell carcinoma (OSCC) remains associated with poor survival, and conventional prognostic indicators such as TNM staging provide limited accuracy. Saliva has emerged as a promising liquid biopsy, but evidence regarding its prognostic role is limited. This review systematically assessed salivary proteomic, transcriptomic, and metabolomic biomarkers with prognostic value for survival and disease progression in OSCC patients. **Methods**: A systematic literature search was performed across PubMed, Scopus, Web of Science, Embase, and Cochrane Library up to 7 June 2025, following PRISMA 2020 and JBI guidelines. Human studies evaluating associations between salivary biomarkers and prognosis in OSCC were included. Risk of bias was assessed with the QUIPS tool. The review protocol was registered on PROSPERO (CRD42024535737). **Results**: Fifteen studies were included, involving 872 OSCC patients and 548 healthy controls. Biomarkers were identified using proteomic (*n* = 9), transcriptomic (*n* = 4), and metabolomic (*n* = 2) approaches. Among the most promising, miR-423-5p was independently associated with shorter disease-free survival (DFS), AKR1B10 levels above 646 pg/mL predicted worse overall survival (OS), and 3-methylhistidine was validated as a metabolomic marker of reduced OS. Additionally, miR-1307-5p correlated with nodal metastasis and chemoresistance, Cyfra 21-1 with recurrence and grade, and a low apoptotic/non-apoptotic salivary microvesicle ratio with poorer outcomes. However, heterogeneity in methods, small sample sizes, and lack of external validation limit clinical applicability. **Conclusions**: Salivary biomarkers show potential as non-invasive tools for prognostic stratification in OSCC. Among the identified candidates, miR-423-5p, AKR1B10, and 3-methylhistidine provide the most robust evidence. Future multicenter, longitudinal studies with standardized protocols and validation are essential.

## 1. Introduction

Oral squamous cell carcinoma (OSCC) represents the 16th most common malignant neoplasm worldwide [1]. Despite advances in diagnosis and therapy, delayed detection remains a major issue, particularly in low- and middle-income countries, resulting in a 5-year survival rate of approximately 20% in advanced-stage cases [2,3].

Timely identification of OSCC is critical for improving patient outcomes and reducing disease-related morbidity. In this context, non-invasive diagnostic strategies such as liquid biopsy are gaining traction as valuable tools for the early detection, prognosis, and longitudinal monitoring of OSCC [4]. Among the various biological specimens investigated, saliva has emerged as a particularly suitable matrix for biomarker analysis due to its non-invasive collection, ease of storage, and potential for repeated sampling [5,6,7].

A wide spectrum of salivary biomarkers—ranging from nucleic acids and proteins to metabolites and lipids—has been explored for their diagnostic value in OSCC. For example, a recent systematic review by Khijmatgar et al. identified Chemerin and matrix metalloproteinase-9 (MMP-9) as key diagnostic biomarkers, alongside Phytosphingosine and Pipecolinic acid for early-stage disease [8]. Additionally, microRNAs such as miRNA-136 have shown high diagnostic accuracy in OSCC, although further research is needed to confirm their clinical utility [9]. Another comprehensive review focused on inflammatory cytokines highlighted elevated levels of interleukin-6 (IL-6), interleukin-8 (IL-8), and tumor necrosis factor-alpha (TNF-α) in patients with OSCC compared to healthy controls or individuals with premalignant lesions [10]. These cytokines also appear to correlate with disease stage and tumor aggressiveness. However, existing literature largely focuses on diagnostic rather than prognostic implications, and studies on genomic and metabolomic salivary biomarkers in the context of OSCC prognosis remain scarce.

While numerous studies have addressed the diagnostic potential of salivary biomarkers in OSCC [11,12,13,14], fewer have examined their prognostic relevance. Identifying biomarkers capable of predicting disease progression, recurrence, or survival outcomes could significantly improve patient stratification and management. Recent systematic reviews have explored the role of salivary biomarkers in OSCC prognosis, but significant methodological and conceptual differences remain compared to the present review. Rengasamy et al. [15] conducted a review focusing exclusively on inflammatory cytokines such as IL-6, IL-8, and TNF-α, without including transcriptomic or metabolomic biomarkers, and without a quantitative synthesis of prognostic data. Similarly, Ravindran et al. [16] provided a broad narrative overview encompassing diagnostic, prognostic, and therapeutic implications of molecular biomarkers in OSCC, but included in vitro and animal studies, lacked formal risk of bias assessment, and did not distinguish biomarkers based on clinical outcome stratification. In contrast, Qin et al. [17] focused their review on salivary miRNAs and cytokines, with emphasis on their diagnostic potential and molecular mechanisms, but did not adopt a systematic approach nor apply inclusion criteria based on prognostic endpoints. Moreover, the review by Qin et al. was limited to studies published within the last three years and did not incorporate proteomic or metabolomic biomarkers, thereby restricting the scope of prognostic insights.

In contrast, the present systematic review is the first to focus exclusively on the prognostic significance of salivary biomarkers in OSCC. The primary objective is to systematically identify and evaluate salivary proteomic, transcriptomic, and metabolomic biomarkers that are significantly associated with clinical outcomes such as disease-free survival (DFS), overall survival (OS), and tumor progression in OSCC patients. The ultimate goal is to determine which biomarkers demonstrate the strongest evidence for prognostic use and to assess the quality and consistency of the current literature.

## 2. Materials and Methods

### 2.1. Focused Question

Which salivary proteomic, transcriptomic, or metabolomic biomarkers are most strongly associated with prognosis—specifically DFS, OS, or tumor progression—in patients with OSCC?

### 2.2. Eligibility Criteria

The inclusion criteria considered for this review were (I) Study design—prospective or retrospective cohort studies, case-control studies, or clinical trials specifically designed to evaluate prognostic associations between salivary biomarkers and clinical outcomes; (II) Population—human participants of any age with a histopathologically confirmed diagnosis of oral squamous cell carcinoma (OSCC); (III) Prognostic factor—salivary biomarkers of proteomic, transcriptomic, or metabolomic origin investigated for their association with patient prognosis; (IV) Outcome—reported data on associations between salivary biomarkers and at least one predefined prognostic endpoint, including TNM stage, histopathological grade, lymph node involvement, local or distant recurrence, disease progression, disease-free survival (DFS), or overall survival (OS); (V) Timing—prognostic outcomes evaluated during treatment, after treatment completion, or at follow-up time points such as 6 months, 1 year, or longer; (VI) Setting—no restrictions regarding clinical setting or geographic location; (VII) Language and publication date—no restrictions on language or date of publication.

The analysis was limited to studies that satisfied all the inclusion criteria, while the exclusion criteria comprised the following aspects: (I) duplicate publications; (II) studies lacking approval from an institutional or national Ethics Committee; (III) case reports or case series; (IV) in vitro studies or laboratory-based experimental research; (V) ex vivo or animal studies; (VI) narrative reviews, scoping reviews, systematic reviews, and meta-analyses; (VII) studies analyzing biomarkers in biological samples other than saliva; (VIII) studies focused solely on diagnostic or therapeutic outcomes, without reporting prognostic associations; (IX) studies involving populations that did not meet the predefined eligibility criteria.

### 2.3. Search Strategy and Study Selection

A three-step search strategy was conducted in accordance with the JBI methodology for systematic reviews of prognostic evidence [18]. First, a preliminary limited search was performed on PubMed (MEDLINE), Scopus, Web of Science (WoS), Embase, and Cochrane Library to identify relevant articles and extract key terms from titles, abstracts, and index terms. Second, a comprehensive and tailored search strategy was developed individually for each database using both keywords and controlled vocabulary (MeSH terms), with combinations related to “oral squamous cell carcinoma,” “saliva,” “biomarkers,” and “prognosis.” Boolean operators were applied as appropriate to enhance search sensitivity. Finally, the reference lists of all included studies and relevant systematic reviews were manually screened to identify any additional eligible publications.

The search strategy was guided by the PICOTS framework (Population, Index prognostic factors, Comparator/Covariates, Outcomes, Timing, Setting), ensuring full alignment with the study’s eligibility criteria and focused objective of identifying salivary biomarkers associated with prognostic outcomes in OSCC (Table 1). The review process followed the Preferred Reporting Items for Systematic Reviews and Meta-Analyses (PRISMA) 2020 guidelines [19], as detailed in Table S1 (Supplementary Materials). No restrictions were applied regarding language or publication date. Table S2 (Supplementary Materials) reports the full, database-specific search strategies used for PubMed (MEDLINE), Scopus, Web of Science (WoS), Embase, and Cochrane Library, including the exact number of records retrieved from each source. The final search was conducted on 7 June 2025.

This review protocol was prospectively registered on the PROSPERO platform (CRD42024535737), it is available at: https://www.crd.york.ac.uk/prospero/display_record.php?ID=CRD42024535737 (accessed on 30 August 2025).

Study selection was performed in two phases by two independent and blinded reviewers (M.Pe. and M.Pa.). In the first phase, titles and abstracts of all retrieved records were screened based on the predefined eligibility criteria, and studies such as narrative reviews, systematic reviews, meta-analyses, book chapters, and irrelevant records were excluded. In the second phase, full texts of potentially eligible articles were independently reviewed to confirm inclusion. Disagreements were resolved through discussion and consensus. When consensus was not reached or the eligibility of a study remained ambiguous, five additional reviewers were consulted independently, and decisions were based on majority agreement. No automation tools were employed during the screening or selection process.

### 2.4. Data Extraction

The purpose of data extraction was to systematically collect relevant information from each included study to enable structured synthesis and comparison of prognostic evidence related to salivary biomarkers in OSCC. Data extraction was independently performed by two reviewers using a standardized spreadsheet specifically developed for this review. The extracted information included study characteristics (first author, year of publication, country), study design, number of participants, characteristics of OSCC populations, salivary biomarkers investigated, analytical techniques used, prognostic outcomes assessed, and key findings. The spreadsheet was updated progressively and reviewed throughout the extraction process.

Data extraction was conducted between April and June 2025, with the final literature search completed on 7 June 2025. All eligible studies published up to that date were included. Due to the qualitative scope of this review and the overall completeness of published data, no attempts were made to contact study authors, in order to ensure consistency and minimize potential response bias.

Discrepancies between reviewers were resolved by discussion and consensus. In particularly ambiguous cases, five additional reviewers were independently consulted, and final decisions were reached by majority agreement. No automation tools or software-assisted extraction platforms were used.

The extracted data were synthesized into structured comparative tables categorized by biomarker type (proteomic, transcriptomic, or metabolomic), study design, and prognostic endpoint (DFS, OS, progression, recurrence). No formal assessment of reporting bias, such as publication bias or selective outcome reporting, was undertaken, due to the absence of meta-analytic pooling. Likewise, the certainty of evidence was not formally graded using tools such as GRADE, in light of the methodological heterogeneity and observational design of the included studies.

### 2.5. Quality Assessment of Included Studies

The methodological quality and risk of bias (RoB) of the included prognostic studies were evaluated using the Quality In Prognosis Studies (QUIPS) tool [20], a validated instrument specifically designed for assessing risk of bias in prognostic factor research. This tool examines six key methodological domains: study participation, study attrition, prognostic factor measurement, outcome measurement, study confounding, and statistical analysis and reporting.

Domain-specific judgments were formulated based on signaling questions and guidance provided by the original developers of the QUIPS tool. The detailed criteria used for each domain are reported in Table S3 (Supplementary Materials), while the interpretation of the individual domains is summarized in Table S4 (Supplementary Materials). In line with the QUIPS methodology, no global summary score or overall rating was assigned to individual studies, to avoid masking important domain-specific biases.

Moreover, Table S5 (Supplementary Materials) lists the full references of the research papers that were excluded from this systematic review, along with clear justifications for their exclusion [21,22,23,24,25,26,27,28,29,30,31,32,33,34,35,36,37,38,39,40]. The most frequent causes were the absence of prognostic assessment, which characterized fourteen studies [21,22,23,24,25,27,28,31,32,36,37,38,39,40] focused mainly on diagnostic accuracy or descriptive comparisons; the use of tumor tissue rather than salivary specimens, reported in four studies [29,33,34,35]; and the inclusion of non-eligible populations, observed in two studies [26,30] involving either non-OSCC patients or mixed head and neck cancer cohorts without separate OSCC analyses.

## 3. Results

**Figure 1:**
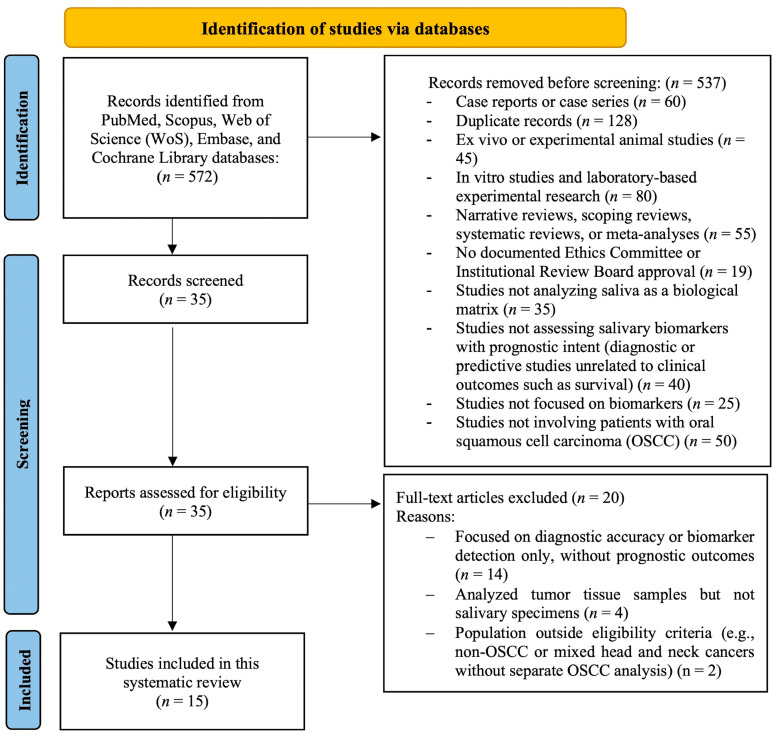
PRISMA 2020 flow diagram of the systematic review process.

The initial database search identified a total of 572 articles from PubMed, Scopus, Web of Science (WoS), Embase, and the Cochrane Library, without restrictions on language or publication year. After the removal of 537 records during the screening phase, the remaining 35 articles were assessed in full text for eligibility. Ultimately, 15 studies met all inclusion criteria and were included in the systematic review. The detailed reasons for study exclusion at each stage of the selection process are illustrated in Figure 1, following the PRISMA 2020 flow diagram.

Among the studies included in this systematic review, ten were cross-sectional studies [41,42,43,44,45,46,47,48,49,50], and five were observational cohort studies [51,52,53,54,55].

The main demographic and clinical characteristics of the study populations, along with TNM staging and eligibility criteria, are summarized in Table 2. This includes the number of OSCC patients and controls, gender distribution, age data, tumor staging or grading, and inclusion/exclusion criteria applied in each study.

Table 3 presents an overview of the study design, analytical techniques, and main salivary biomarkers investigated across the included studies. The table also summarizes the comparisons between OSCC patients and controls, as well as any reported prognostic associations, including disease-free survival and overall survival where available.

### 3.1. Risk of Bias Assessment

The risk of bias assessment using the QUIPS tool revealed a generally acceptable methodological quality across the included studies, although some weaknesses were noted in specific domains. In terms of study participation, most studies provided an adequate description of the population of interest, baseline sample, and recruitment procedures. Twelve out of fifteen studies, including those by Bu et al. [42], Winck et al. [43], Malhotra et al. [44], Pathiyil et al. [45], Ko et al. [46], Wang et al. [51], Zhong et al. [52], Romani et al. [53], Ishikawa et al. [54], Patel et al. [47,49], Shabbir et al. [48], and Premkumar et al. [50], were rated as having low risk of bias. In contrast, Aziz et al. [41] and Hema Shree et al. [55] were judged to have moderate risk due to partially reported or unclear information regarding the recruitment framework and setting.

Study attrition emerged as the domain with the highest frequency of methodological limitations. Most studies failed to provide sufficient details on follow-up, dropouts, or comparisons between completers and non-completers. Consequently, thirteen studies were rated as having moderate risk of bias, while Hema Shree et al. [55] was judged at high risk due to the complete lack of information regarding attrition, despite its longitudinal design. Ishikawa et al. [54] was the only study in which the attrition domain was judged at low risk of bias, as all patients were followed over time and no substantial loss to follow-up was reported. This limitation has important implications for prognostic research: inadequate reporting of attrition undermines confidence in the stability of biomarker–outcome associations and raises the possibility that observed prognostic effects may be biased by selective loss of participants with unfavorable prognoses. At the level of this systematic review, the recurrent issues with attrition reduce the strength of the cumulative evidence, particularly for biomarkers evaluated in single studies or without independent replication.

Prognostic factor measurement was consistently well-reported across the studies. All studies clearly defined the biomarker assessed, used valid and reliable analytical methods, and applied the same measurement protocols to all participants. Although no study explicitly addressed missing data or imputation strategies—resulting in all being rated as “unsure” on that sub-item—fourteen studies were ultimately considered at low risk of bias in this domain, with only Aziz et al. [41] rated as moderate due to partial methodological reporting. This consistency supports the internal validity of individual biomarker assessments but does not mitigate concerns regarding external reproducibility.

Outcome measurement was generally well-handled. All studies provided clear definitions of outcomes, and most used valid, reliable methods consistently across participants. The only exception was Hema Shree et al. [55], which received a moderate risk rating due to partially reported methodology in outcome measurement and inconsistencies between the gold-enhanced and standard ELISA analysis phases.

Study confounding was more variably addressed. While most studies acknowledged potential confounding factors, such as tumor stage, grade, or treatment status, these were not consistently defined, measured, or statistically adjusted for. Only Bu et al. [42] was judged at low risk, while most others, including Ishikawa et al. [54], were rated as moderate. In Ishikawa et al. [54], although some confounders such as stage and treatment were considered in the multivariate Cox model, other relevant variables were either not clearly defined or not fully addressed in the design phase. This lack of consistency in handling confounders may have inflated or attenuated biomarker–outcome associations, and for this review it implies that the strength of the prognostic claims should be interpreted with caution, particularly in the absence of standardized adjustments across studies.

Finally, statistical analysis and reporting were generally appropriate across the dataset. Most studies used adequate models for their design, clearly presented results, and avoided selective reporting. All studies, including Ishikawa et al. [54], received a low risk of bias rating in this domain, with the exception of Patel et al. [47] and Hema Shree et al. [55], which were rated moderate.

### 3.2. Results of Syntheses

A total of 15 studies investigating the prognostic potential of salivary biomarkers in patients with oral squamous cell carcinoma (OSCC) were included in this review. The study designs consisted mainly of cross-sectional analyses (10/15, 67%) and five cohort studies (33%), two of which implemented longitudinal salivary sampling protocols [54,55]. These studies, published between 2015 and 2025, originated predominantly from India (8/15, 53%) [44,45,47,49,50,55], followed by Taiwan [46,51], China [42,52], Pakistan [41,48], Brazil [43], Italy [53], and Japan [54], reflecting a notable concentration of research in Asian contexts. Collectively, the studies included 872 OSCC patients and 548 healthy controls, although demographic data for the control groups were not consistently reported. To provide a more structured overview of the heterogeneous evidence, the results are presented as a subgroup synthesis by biomarker class.

#### 3.2.1. Proteomic Biomarkers

Nine studies adopted proteomic approaches [41,43,44,46,48,50,51,52,55], focusing on inflammatory cytokines (e.g., IL-10, IL-13, TNF-α), cytoskeletal and apoptotic proteins (e.g., Cyfra 21-1, 14-3-3, S100P, survivin), and metabolic enzymes (e.g., AKR1B10, Cathepsin B). Significant differences in salivary levels between OSCC patients and controls were reported in nearly all studies (*p* < 0.05 to <0.001). Most proteomic markers were linked to surrogate endpoints, including tumor stage and histological grade [44,48,55]. However, some also demonstrated prognostic associations with survival: Ko et al. [46] reported that AKR1B10 levels exceeding 646 pg/mL were a prognostic factor for reduced overall survival (OS), while Zhong et al. [52] showed that a lower apoptotic/non-apoptotic microvesicle ratio correlated with poorer OS.

#### 3.2.2. Transcriptomic Biomarkers

Four studies investigated transcriptomic profiles [42,47,49,53], consistently implicating microRNAs and mRNA transcripts. Surrogate endpoints included recurrence, lymph node metastasis, and epithelial–mesenchymal transition, as demonstrated for miR-1307-5p [47] and transgelin mRNA/miR-145-5p [49]. Importantly, Romani et al. [53] identified 25 differentially expressed salivary miRNAs, of which seven were significantly associated with disease-free survival (DFS). Among them, miR-423-5p emerged as an independent prognostic factor for shorter DFS (HR = 2.58; 95% CI: 1.42–4.71; *p* = 0.002), and its levels significantly declined after tumor resection (*p* < 0.001). Thus, transcriptomic biomarkers provided both indirect and direct evidence of prognostic utility.

#### 3.2.3. Metabolomic Biomarkers

Two studies implemented metabolomic approaches [45,54]. Pathiyil et al. [45] assessed metabolite changes primarily in relation to tumor stage, whereas Ishikawa et al. [54] conducted a prospective CE-TOFMS study that identified 3-methylhistidine as an independent prognostic factor for reduced OS, confirmed in both training and validation cohorts. In contrast, N-acetylglucosamine was associated with shorter DFS in the training set but lost significance in validation, underscoring the need for replication.

#### 3.2.4. Comparative Trends

Across these biomarker classes, no omic category clearly outperformed the others. Proteomic studies identified a wide range of candidate molecules but showed greater heterogeneity and lacked external validation. Transcriptomic analyses yielded the most reproducible associations, especially with DFS and recurrence, while metabolomic evidence, though sparse, provided validated prospective insights. Together, these subgroup findings underscore the promise of salivary biomarkers for OSCC prognosis but also the urgent need for multicenter studies with standardized protocols and external validation.

Most studies recruited untreated OSCC patients and healthy controls matched by age and sex. However, only 5 studies (33%) [41,47,48,53,55] provided detailed demographic information for controls, and inclusion/exclusion criteria were inconsistently reported. This variability may introduce heterogeneity in the assessment of control comparability and affect the interpretability of prognostic findings.

Prospective and cohort studies employed more advanced and standardized analytical platforms, such as CE-TOFMS [54], reverse transcription quantitative PCR (RT-qPCR) [49,53], two-dimensional difference gel electrophoresis with MALDI-TOF-MS [51], and gold nanoparticle-enhanced ELISA [55], thereby enhancing internal validity. Nonetheless, none of the included studies conducted external validation in independent patient cohorts, which remains a critical limitation for the clinical implementation of these biomarkers.

#### 3.2.5. Prognostic Endpoints Versus Surrogate Measures

Among the fifteen included studies, only a minority directly evaluated survival outcomes, while the majority focused on surrogate measures such as tumor stage, histological grade, recurrence, or related biological phenotypes. Four studies (27%) provided the strongest prognostic evidence by assessing disease-free survival (DFS) and/or overall survival (OS). These included miR-423-5p [53] for DFS, AKR1B10 [46] and the apoptotic/non-apoptotic microvesicle ratio [52] for OS, and 3-methylhistidine [54] for OS, with internal validation.

In contrast, the remaining eleven studies (73%) primarily investigated surrogate measures. Examples include IL-10 and IL-13 [41], survivin [43], Cyfra 21-1 [44], metabolite panels [45], miR-1307-5p [47], Cathepsin B [48], S100P and 14-3-3 [50], proteomic profiles assessed by Wang et al. [51], IL-10 and TNF-α evaluated by Hema Shree et al. [55], transgelin mRNA and miR-145-5p [49], and additional transcriptomic markers explored by Bu et al. [42]. While surrogate markers provide valuable insights into tumor biology and disease aggressiveness, their prognostic validity is indirect compared with survival endpoints. This distinction emphasizes the preliminary nature of most current findings and highlights the need for biomarkers to be validated against robust clinical outcomes.

## 4. Discussion

This systematic review assessed the prognostic value of salivary biomarkers—derived from proteomic, transcriptomic, and metabolomic approaches—in patients with oral squamous cell carcinoma (OSCC). The primary aim was to identify biomarkers associated with clinical outcomes such as disease-free survival (DFS), overall survival (OS), and tumor progression. Among the 15 eligible human studies, a wide range of molecular platforms and biomarker classes were explored, reflecting growing interest in saliva as a non-invasive tool for oncological prognostication.

Proteomic biomarkers were the most frequently investigated. Several studies identified proteins such as Cyfra 21-1, Cathepsin B, AKR1B10, IL-10, IL-13, and TNF-α as being associated with tumor burden, histological grade, or recurrence risk. In particular, Cyfra 21-1 was correlated with tumor differentiation and recurrence, while AKR1B10 emerged as a significant predictor of poor OS when levels exceeded 646 pg/mL in early-stage OSCC. Other proteins, including survivin and S100P, were implicated in cell survival and proliferation pathways. Despite these associations, many proteomic studies lacked prospective design or external validation, limiting their generalizability.

Transcriptomic biomarkers demonstrated particularly strong prognostic potential. Romani et al. showed that elevated salivary miR-423-5p levels were independently associated with shorter DFS and declined significantly after surgery, suggesting both diagnostic and prognostic utility. Patel et al. found miR-1307-5p to be associated with nodal metastasis and chemoresistance, while a downregulation of miR-140-5p, miR-143-5p, and miR-145-5p was indicative of epithelial–mesenchymal transition and tumor aggressiveness [47]. These transcriptomic signatures support the concept that salivary RNA can reflect key oncogenic processes relevant to prognosis.

Metabolomic studies, though fewer, contributed novel insights. Ishikawa et al. identified 3-methylhistidine as a salivary metabolite independently associated with poor prognosis (reduced OS), confirmed in both training and validation cohorts. This biomarker outperformed others such as N-acetylglucosamine and 5-hydroxylysine, which failed to maintain significance upon validation. These findings suggest that salivary metabolomics, when applied with rigorous methodology and internal validation, may provide reproducible prognostic indicators.

Six of the fifteen included studies directly investigated survival outcomes (DFS and/or OS), with variable levels of statistical rigor and validation. miR-423-5p, AKR1B10, 3-methylhistidine, and the apoptotic/non-apoptotic salivary microvesicle ratio were the most consistent and clinically promising predictors across different omic platforms. However, the absence of external validation cohorts and small sample sizes were common limitations.

In addition to biomarker-level findings, several methodological issues emerged. The predominance of cross-sectional designs (67%) limits inference on temporal changes and disease progression. Few studies reported comprehensive demographic information or clearly defined control group criteria, reducing the interpretability of between-group comparisons. Furthermore, heterogeneity in analytical platforms—ranging from ELISA and LC-MS/MS to RT-qPCR and CE-TOFMS—complicates direct comparison and reproducibility across studies.

No study conducted external validation or tested biomarker panels in a clinical trial setting, a necessary step before clinical application. The lack of standardization in pre-analytical protocols, sampling timing, and normalization methods further restricts clinical translation. Moreover, although most included studies demonstrated acceptable methodological quality in several QUIPS domains, recurrent issues with attrition and incomplete handling of confounding substantially reduce confidence in prognostic validity. As a result, the associations observed for promising biomarkers—such as miR-423-5p, AKR1B10, and 3-methylhistidine—should be regarded as preliminary and interpreted with caution until replicated in larger, prospectively designed multicenter cohorts with complete follow-up and standardized adjustment for confounders.

Despite these limitations, this review identifies several salivary biomarkers with strong potential for non-invasive prognostic stratification in OSCC. Notably, miR-423-5p, AKR1B10, and 3-methylhistidine emerged as the most robust candidates based on current evidence. These biomarkers could complement traditional prognostic tools—such as TNM staging and histopathological grading—by offering dynamic, real-time insights into tumor behavior. Their non-invasive nature makes them suitable for repeated sampling and longitudinal monitoring, supporting personalized follow-up strategies.

Future research should prioritize validation of these biomarkers in external cohorts and in prospective studies, integration into multivariate models alongside clinical variables, and exploration of composite biomarker panels across omic layers. Moreover, studies should investigate how salivary biomarkers might guide therapeutic decision-making and surveillance intensity, particularly in early-stage disease or resource-limited settings.

Ultimately, validated salivary biomarkers may represent a practical and accessible adjunct to current prognostic assessment in OSCC, enabling tailored patient management and improved outcomes through earlier identification of high-risk individuals.

## 5. Conclusions

This systematic review highlights the potential role of salivary biomarkers as non-invasive prognostic tools in patients with oral squamous cell carcinoma (OSCC), considering the ease, considering the ease of collection and non-invasiveness in a purely clinical context. Among the considered studies, miR-423-5p, AKR1B10, and 3-methylhistidine demonstrated the strongest and most reproducible associations with survival outcomes, particularly disease-free survival and overall survival. Other biomarkers, such as miR-1307-5p and Cyfra 21-1, were associated with disease progression and tumor burden, although validation was limited. Further prospective studies are required to increase current evidence that is based mainly on cross-sectional studies.

## Figures and Tables

**Table 1:** PICOTS model followed in this systematic review.

Domain	Description
P—Population	Adults diagnosed with OSCC, regardless of tumor site, stage, or histologic grading
I—Index Prognostic Factors	Salivary biomarkers of proteomic, transcriptomic, and metabolomic nature, assessed for prognostic significance
C—Comparator/Covariates	Healthy individuals or OSCC patients with early-stage disease (stage I–II), low-grade tumors (grade I), or absence of lymph node and distant metastasis. Reported covariates included age, gender, tobacco or alcohol use, TNM stage, and histological grade
O—Outcomes of Interest	Prognostic outcomes included tumor stage at diagnosis, histological grade, presence of lymph node involvement, presence of distant metastases, disease progression, local recurrence, OS, and DFS
T—Timing (Prognostication Time and Follow-up Period)	At the time of OSCC diagnosis or before initiation of treatment. When available, outcome data were reported during treatment, immediately after treatment completion, at 6 months, 1 year, or at the longest follow-up time specified by the study authors
S—Setting	Clinical studies conducted in hospital-based or university-based research settings, without restriction by geographic location

Abbreviations: DFS, Disease-Free Survival; OS, Overall Survival; OSCC, Oral Squamous Cell Carcinoma; TNM, Tumor Node Metastasis.

**Table 2:** Demographic and clinical characteristics of OSCC patients and controls in included studies evaluating salivary prognostic biomarkers.

Authors, Year, and Country of Publication	N° of Patients and Women (%)	N° of Controls and Women (%)	Mean Age ± SD and/or Range (Years) of Patients	TNM Staging and/or Grading (N° of Patients)	Inclusion (IC) and Exclusion Criteria (EC)
Aziz et al., 2015, Pakistan [41]	30; 6 (20%)	33; 6 (18.2%)	50.33 ± 11.77	Grade I: 20 Grade II: 5 Grade III: 5	IC: histopathologically confirmed untreated OSCC; healthy, age- and gender-matched controls; EC: any oral or systemic illness (including periodontal disease).
Bu et al., 2015, Cina [42]	78; 30 (38.5%)	N.R.	N.R.	Stage I-II: 46 Stage III-IV: 32	IC: pathologically confirmed OSCC, no prior treatment before sample collection; EC: N.R.
Winck et al., 2015, Brasil [43]	24; 3 (12.5%)	10; N.R.	N.R.	Grade I: 5 Grade II: 8 Grade III: 5 Grade N.R. for 6 patients	IC: N.R. EC: N.R.
Malhotra et al., 2016, India [44]	50; N.R.	50; N.R.	N.R.	Grade I: 27 Grade II: 23	IC: histologically diagnosed OSCC and healthy controls; EC: history of drugs with anticholinergic effect.
Pathiyil et al., 2017, India [45]	20; N.R.	20; N.R.	N.R.	N.R.	IC: untreated cases of OSCC, healthy controls; EC: patients with previous history of malignancy and treatments and with systemic diseases.
Ko et al., 2018, Taiwan [46]	86; 10 (11.6%)	35; N.R.	N.R.	Stage I-II: 70 Stage III-IV: 16	IC: untreated OSCC patients and healthy controls; EC: patients with prior malignancy, metastasis, recurrence, or preoperative treatment.
Patel et al., 2022, India [47]	21; 0 (0%)	15; 0 (%)	48 ± 8.3	Stage I/II: 8 Stage III/IV: 13	IC: diagnosis of OSCC, history of smokeless tobacco, healthy controls; EC: benign leukoplakia, HIV, HBsAg, HPV, COVID-19 positivity, pediatric patients, samples needed for further histopathological diagnosis, disease-oriented complications
Shabbir et al., 2022, Pakistan [48]	60; 17 (28.3%)	20; 8 (40%)	18–65	Grade I: 20 Grade II: 20 Grade III: 20	IC: OSCC cases and healthy controls; EC: patients with underlying systemic illness, controls with a history of smoking.
Patel et al., 2023, India [49]	23; N.R.	21; N.R.	N.R.	N.R.	IC: untreated cases of OSCC, healthy controls; EC: leucoplakia, HIV, HBsAg, HPV, COVID-19 infected patients, pediatric patients.
Premkumar et al., 2023, India [50]	38; N.R.	38; N.R.	25–80	Grade I: 18 Grade II: 10 Grade III: 10	IC: 25–80 years aged patients diagnosed with OSCC and healthy controls; EC: patients with previous treatments and malignancy history, patients with autoimmune diseases.
Wang et al., 2018, Taiwan [51]	116; N.R.	65; N.R.	N.R.	Early stage: 35 Late stage: 81	IC: OSCC patients who did not receive radiation or chemotherapy and healthy controls; EC: patients with occurrence of synchronous or metachronous primary cancer and who failed to receive the adjuvant therapy when indicated.
Zhong et al., 2019, China [52]	65; N.R.	42; N.R.	N.R.	N.R.	IC: OSCC patients free from any other systemic illness and healthy controls; EC: N.R.
Romani et al., 2021, Italy [53]	89; 27 (30.3%)	58; 20 (34.5%)	24–91	Grade I: 19 Grade II: 49 Grade III: 20 Grade N.R. for 1 patient.	IC: primary OSCC cases and healthy controls; EC: N.R.
Ishikawa et al., 2022, Japan [54]	72; 34 (47.2%)	N.R.	23–94	Stage 0: 3 (4.2%) Stage I: 24 (33.3%) Stage II: 14 (19.4%) Stage III: 13 (18.1%) Stage IV: 18 (25.0%)	IC: diagnosed OSCC, treated with curative intent (surgery or chemoradiotherapy); informed opt-out consent; EC: Non-curative treatment (palliative/symptomatic); declined participation (none did).
Hema Shree et al., 2025, India [55]	40; 16 (40%)	10; 5 (50%)	50–60	N.R.	IC: OSCC patients undergoing longitudinal salivary sampling for TNF-α analysis; EC: patients with recent food intake, oral hygiene procedures, or interfering oral conditions.

Abbreviations: COVID-19, coronavirus disease-19; EC, exclusion criteria; HBsAg, hepatitis B surface antigen; HIV, human immunodeficiency virus; HPV, human papillomavirus; IC, inclusion criteria; N.R., not reported; OSCC, oral squamous cell carcinoma; TNF-α, Tumor Necrosis Factor alpha.

**Table 3:** Summary of clinical studies evaluating the prognostic value of salivary biomarkers in OSCC.

Authors, Year, and Country of Publication	Study Design and Aim	Sample Analysis	Biomarkers	OSCC vs. Controls	Prognostic Association	Disease-Free Survival	Overall Survival
Aziz et al., 2015, Pakistan [41]	Cross-sectional study aimed to evaluate the importance of immunosuppressive cytokines as prospective salivary biomarkers of OSCC	xMAP	IL-4, IL-10, IL-13, IL-1RA (proteomic)	IL-10 ↑ (*p* = 0.004), IL-13 ↑ (*p* = 0.010), IL-1RA ↑ in PDT vs. MDT, WDT, and controls (*p* < 0.002)	Elevated salivary levels of IL-4, IL-10, IL-13, and IL-1RA in OSCC patients vs. controls; IL-1RA significantly higher in PDT vs. MDT and WDT, suggesting involvement of these cytokines in tumor progression and immune evasion	N.R.	N.R.
Bu et al., 2015, Cina [42]	Cross-sectional study to assess salivary transgelin mRNA in OSCC	Real-time PCR	Transgelin mRNA (transcriptomic)	↑ in OSCC vs. HC (*p* < 0.01); ↑ in ETsT, ENsT, ETNMsT, PDT, ENEPT (*p* < 0.05)	Higher salivary transgelin mRNA levels associated with advanced tumor stage (ETsT), nodal stage (ENsT), metastatic stage (ETNMsT), and PDT, indicating correlation with aggressive disease phenotype	N.R.	N.R.
Winck et al., 2015, Brasil [43]	Cross-sectional comparative proteomic profiling of whole saliva from OSCC patients and healthy controls to identify diagnostic and prognostic markers	Shotgun proteomics (LC-MS/MS)	14-3-3 protein zeta/delta, Annexin A1, S100A9 (proteomic)	14-3-3 protein ↑ in OSCC (*p* < 0.05); Annexin A1 and S100A9 ↓ in OSCC (*p* < 0.01)	14-3-3 protein associated with tumor cell proliferation and survival; Annexin A1 downregulation linked to loss of anti-inflammatory and pro-apoptotic control; S100A9 reduction may indicate impaired immune response—together suggesting potential markers of poor prognosis	N.R.	N.R.
Malhotra et al., 2016, India [44]	Cross-sectional study to evaluate Cyfra 21-1 levels and their correlation with tissue CK19 mRNA	ECLIA	Cyfra 21-1 (proteomic)	↑ in OSCC vs. HC (*p* < 0.001); ↑ in grade II vs. grade I; ↑ in recurrence cases (*p* < 0.005)	Higher salivary Cyfra 21-1 levels correlated with increased tumor grade (grade II > I), recurrence, and CK19 mRNA tissue expression, supporting its role in predicting recurrence and histological aggressiveness	N.R.	N.R.
Pathiyil et al., 2017, India [45]	Cross-sectional study investigating the utility of salivary LDH as a prognostic marker in OSCC	Spectrophotometry at 340 nm	LDH (metabolomic)	LDH significantly ↑ in OSCC vs. HC (457.06 ± 88.93 IU/L vs. 178.35 ± 120.54 IU/L; *p* < 0.001)	Salivary LDH significantly ↑ one month post-surgery vs. preoperative levels (*p* < 0.001); potential marker of disease burden and recurrence	N.R.	N.R.
Ko et al., 2018, Taiwan [46]	Cross-sectional study to assess salivary AKR1B10 as screening and prognostic marker	ELISA	AKR1B10 (proteomic)	↑ in OSCC vs. HC (*p* < 0.001); ↑ in ETsT and ETNMsT (*p* < 0.05)	AKR1B10 levels increased in OSCC vs. controls and in advanced tumor (ETsT) and nodal stages (ETNMsT); levels > 646 pg/mL in early-stage tumors (LTsT) predicted shorter survival, supporting its prognostic significance	N.R.	Lower OS associated with AKR1B10 levels > 646 pg/mL in early-stage OSCC
Patel et al., 2022, India [47]	Cross-sectional study to identify OSCC-specific salivary exosomal miRNAs	TEM, RNA extraction, miRNA sequencing	miR-1307-5p (transcriptomic)	↑ in OSCC vs. HC (493 vs. 23 reads); ↑ in ETNMsT, ENsT, chemoresistant cases (*p* < 0.05)	Overexpression of miR-1307-5p in OSCC saliva correlated with advanced nodal stage (ETNMsT), extranodal spread (ENsT), and chemoresistance, indicating prognostic value in aggressive and treatment-resistant OSCC	N.R.	N.R.
Shabbir et al., 2022, Pakistan [48]	Cross-sectional study evaluating salivary Cathepsin B levels among patients with different OSCC histological grades	ELISA	Cathepsin B (proteomic)	Salivary Cathepsin B significantly ↑ in OSCC vs. HC (detected in 70% OSCC vs. 15% controls; *p* < 0.001); ↑ with increasing tumor burden	Cathepsin B levels reflect histological grade and tumor progression; potential for prognostic stratification	N.R.	N.R.
Patel et al., 2023, India [49]	Cross-sectional study aimed to identify differentially expressed miRNAs in OSCC and validate their diagnostic/prognostic potential in saliva	Small RNA-Seq, qRT-PCR, transcriptome analysis	miR-140-5p, miR-143-5p, miR-145-5p (transcriptomic)	miRNAs differentially expressed in OSCC vs. HC (*p* ≤ 0.05); miR-140-5p, miR-143-5p, miR-145-5p significantly ↓ in OSCC (validated via qPCR; *p* < 0.01).	Downregulation of the 3-miRNA panel associated with increased tumor aggressiveness, EMT, and poor prognosis	N.R.	N.R.
Premkumar et al., 2023, India [50]	Comparative cross-sectional study investigating survivin expression in salivary secretions of OSCC patients with different histological grades	ELISA	Survivin (proteomic)	Mean salivary survivin significantly ↑ in OSCC vs. HC (9.51 ± 2.9 vs. 2.69 ± 1.2 pg/mL; *p* < 0.001); ↑ with increasing tumor grade (*p* < 0.001).	Elevated survivin levels strongly correlated with tumor grade; higher in poorly differentiated OSCC, suggesting worse prognosis	N.R.	N.R.
Wang et al., 2018, Taiwan [51]	Cohort proteomic study assessing salivary protein biomarkers for early OSCC detection using 2D-DIGE and MS	2D-DIGE, MALDI-TOF-MS, Western blot	Mac-2 binding protein, S100A7, S100P, CST1, KPNA2 (proteomic)	Multiple proteins significantly ↑ in OSCC vs. HC (fold change > 2; *p* < 0.05); KPNA2 significantly ↑ in OSCC (*p* < 0.01)	CST1, S100P, and KPNA2 overexpression associated with proliferation, nuclear transport dysregulation, and poor prognosis	N.R.	N.R.
Zhong et al., 2019, China [52]	Cohort study evaluating the association between salivary microvesicles (SMVs) and clinicopathological features and prognosis in OSCC.	Flow cytometry, TEM, immunohistochemistry	Salivary SMVs (proteomic)	SMVs significantly ↑ in OSCC vs. HC and oral ulcer (*p* < 0.001); ↑ in OSCC with lymph node metastasis and advanced clinical stage (*p* < 0.01)	Lower apoptotic/non-apoptotic SMV ratio associated with higher pathological grade and reduced overall survival (*p* < 0.01)	N.R.	Overall survival significantly reduced in patients with a low apoptotic/non-apoptotic SMV ratio (*p* = 0.001).
Romani et al., 2021, Italy [53]	Cohort genome-wide salivary miRNA study profiling to assess diagnostic and prognostic potential in OSCC patients	Microarray, RT-qPCR	miR-423-5p, miR-106b-5p, miR-193b-3p (transcriptomic)	Multiple miRNAs differentially expressed in OSCC vs. HC (*p* ≤ 0.05); miR-423-5p ↑ in OSCC, ↓ post-surgery (*p* < 0.001).	High salivary miR-423-5p is an independent predictor of shorter DFS in multivariate analysis (*p* < 0.05)	High miR-423-5p was an independent predictor of shorter DFS in multivariate analysis (HR = 2.58; 95% CI: 1.42–4.71; *p* = 0.002).	N.R.
Ishikawa et al., 2022, Japan [54]	Prospective observational study aiming to identify salivary metabolomic biomarkers with prognostic value in OSCC	CE-TOFMS	3-methylhistidine, 5-hydroxylysine, N-acetylglucosamine, proline, creatinine (metabolomic)	Not evaluated (no healthy control group included)	3-methylhistidine and 5-hydroxylysine associated with OS in training set; only 3-methylhistidine retained significance in validation set	N-acetylglucosamine significant for DFS in training set, not confirmed in validation set	3-methylhistidine associated with reduced OS in both training and validation sets
Hema Shree et al., 2025, India [55]	Cohort study aimed at assessing the efficacy of a gold nanoparticle-enhanced ELISA for the detection of TNF-α in saliva of OSCC patients, and evaluating its prognostic value for survival	ELISA and gold nanoparticle-enhanced ELISA	TNF-α (proteomic)	Mean salivary Tumor Necrosis Factor alpha (TNF-α) was significantly ↑ in OSCC patients compared to healthy controls, both with conventional ELISA (47.52 ± 20.23 vs. 10.13 ± 3.07 pg/mL; *p* < 0.001) and gold nano-enhanced ELISA (57.63 ± 24.99 vs. 12.07 ± 3.66 pg/mL; *p* < 0.001). TNF-α levels were also found to ↑ progressively with increasing tumor grade and clinical stage (*p* < 0.001).	Although TNF-α levels were higher in more advanced stages and tumor grades, the Cox proportional hazards model showed no significant association between TNF-α levels and overall survival (*p* = 0.653). Therefore, its prognostic value remains uncertain in this dataset.	N.R.	Kaplan-Meier analysis showed variable survival trends depending on the method and TNF-α levels. The gold nano-enhanced method suggested better survival in patients with TNF-α above the cutoff after 9 months, but no statistically significant differences were confirmed (log-rank *p* = 0.78).

Abbreviations: ↑, increase; ↓, reduction; AKR1B10, Aldo-Keto Reductase Family 1 Member B10; CE-TOFMS, Capillary Electrophoresis Time-Of-Flight Mass Spectrometry; CK19, Cytokeratin 19; Cyfra 21-1, Cytokeratin 19 Fragment Antigen 21-1; DFS, Disease-Free Survival; ECLIA, Electrochemiluminescence Immunoassay; ELISA, Enzyme-Linked Immunosorbent Assay; ENEPT, Extranodal Extension Positive Tumors; ENsT, Extranodal Stage Tumors; ETNMsT, Extra-Tumoral and Nodal Metastatic Stage Tumors; ETsT, Extra-Tumoral Stage Tumors; HC, Healthy Controls; IL-1RA, Interleukin-1 Receptor Antagonist; IL-4, IL-10, IL-13, Interleukins 4, 10, and 13; IU/L, International Units per Liter; KPNA2, Karyopherin Subunit Alpha 2; LDH, Lactate Dehydrogenase; LTsT, Low Tumor Stage Tumors; MALDI-TOF-MS, Matrix-Assisted Laser Desorption/Ionization Time-of-Flight Mass Spectrometry; miRNA/miR, MicroRNA; MS, Mass Spectrometry; N.R., Not Reported; OS, Overall Survival; OSCC, Oral Squamous Cell Carcinoma; PCR/qRT-PCR/RT-qPCR, (Quantitative) Reverse Transcription Polymerase Chain Reaction; PDT, Poorly Differentiated Tumors; S100A7, S100A9, S100P, Calcium-binding proteins of the S100 family; SMVs, Salivary Microvesicles; TEM, Transmission Electron Microscopy; TNF-α, Tumor Necrosis Factor alpha; WDT, Well Differentiated Tumors; xMAP, Multiplexed Bead-based Immunoassay Technology; 2D-DIGE, Two-Dimensional Difference Gel Electrophoresis.

## Data Availability

Upon request to the corresponding author, the data are available for use.

## Supplementary Materials

The following supporting information can be downloaded at: https://www.mdpi.com/article/10.3390/dj13100479/s1, Table S1: PRISMA 2020 Checklist; Table S2: Search strategies used for each database and number of records retrieved; Table S3: Criteria for judging risk of bias according to the QUIPS tool; Table S4: Risk of bias analysis of prognostic studies using the QUIPS tool; Table S5: Summary table of studies excluded in this systematic review.

## Abbreviations

The following abbreviations are used in this manuscript:

AKR1B10	Aldo-Keto Reductase Family 1 Member B10
CE-TOFMS	Capillary Electrophoresis Time-Of-Flight Mass Spectrometry
CK19	Cytokeratin 19
CST1	Cystatin SN
DFS	Disease-Free Survival
ECLIA	Electrochemiluminescence Immunoassay
ELISA	Enzyme-Linked Immunosorbent Assay
EMT	Epithelial–Mesenchymal Transition
ENEPT	Extranodal Extension-Positive Tumors
ENsT	Extranodal Stage Tumor
ETNMsT	Early Tumor With Nodal And Metastatic Spread
ETsT	Early Tumor Stage Tumor
HC	Healthy Controls
HR	Hazard Ratio
IL-10	Interleukin-10
IL-13	Interleukin-13
IL-1RA	Interleukin-1 Receptor Antagonist
KPNA2	Karyopherin Subunit Alpha 2
LC-MS/MS	Liquid Chromatography Tandem Mass Spectrometry
LTsT	Low Tumor Stage Tumor
Mac-2	Galectin-3 Binding Protein
MALDI-TOF-MS	Matrix-Assisted Laser Desorption Ionization Time-Of-Flight Mass Spectrometry
miR	Microrna
N.R.	Not Reported
OS	Overall Survival
OSCC	Oral Squamous Cell Carcinoma
PDT	Poorly Differentiated Tumor
qRT-PCR	Quantitative Reverse Transcription Polymerase Chain Reaction
RNA-Seq	RNA Sequencing
RT-qPCR	Reverse Transcription Quantitative Polymerase Chain Reaction
S100A7	S100 Calcium-Binding Protein A7
S100A9	S100 Calcium-Binding Protein A9
S100P	S100 Calcium-Binding Protein P
SMVs	Salivary Microvesicles
TNF-α	Tumor Necrosis Factor Alpha
