# Supplementary material for: Salivary Biomarkers as Prognostic Tools in Oral Squamous Cell Carcinoma: A Systematic Review of Survival and Progression Outcomes
Source: Dent J (Basel). 2025 Oct 17;13(10):479. doi: 10.3390/dj13100479 (PMC12563148; doi:10.3390/dj13100479)
Supplement: Supplementary file 1 [file dentistry-13-00479-s001.zip › dentistry-3876886-supplementary.pdf]

Table S1. PRISMA 2020 Checklist [1].

Section and Topic	Item #	Checklist item	Location where item is reported
**TITLE**			
Title	1	Identify the report as a systematic review.	1
**ABSTRACT**			
Abstract	2	See the PRISMA 2020 for Abstracts checklist.	2
**INTRODUCTION**			
Rationale	3	Describe the rationale for the review in the context of existing knowledge.	2-4
Objectives	4	Provide an explicit statement of the objective(s) or question(s) the review addresses.	4
**METHODS**			
Eligibility criteria	5	Specify the inclusion and exclusion criteria for the review and how studies were grouped for the syntheses.	4-5
Information sources	6	Specify all databases, registers, websites, organisations, reference lists and other sources searched or consulted to identify studies. Specify the date when each source was last searched or consulted.	5
Search strategy	7	Present the full search strategies for all databases, registers and websites, including any filters and limits used.	Supplementary Table 2
Selection process	8	Specify the methods used to decide whether a study met the inclusion criteria of the review, including how many reviewers screened each record and each report retrieved, whether they worked independently, and if applicable, details of automation tools used in the process.	5
Data collection process	9	Specify the methods used to collect data from reports, including how many reviewers collected data from each report, whether they worked independently, any processes for obtaining or confirming data from study investigators, and if applicable, details of automation tools used in the process.	6
Data items	10a	List and define all outcomes for which data were sought. Specify whether all results that were compatible with each outcome domain in each study were sought (e.g. for all measures, time points, analyses), and if not, the methods used to decide which results to collect.	6
	10b	List and define all other variables for which data were sought (e.g. participant and intervention characteristics, funding sources). Describe any assumptions made about any missing or unclear information.	6
Study risk of bias	11	Specify the methods used to assess risk of bias in the included studies, including details of the tool(s) used, how many reviewers assessed each study and whether they worked independently, and if applicable, details of automation tools	6-7

Section and Topic	Item #	Checklist item	Location where item is reported
assessment		used in the process.	
Effect measures	12	Specify for each outcome the effect measure(s) (e.g. risk ratio, mean difference) used in the synthesis or presentation of results.	Not applicable (qualitative synthesis)
Synthesis methods	13a	Describe the processes used to decide which studies were eligible for each synthesis (e.g. tabulating the study intervention characteristics and comparing against the planned groups for each synthesis (item #5)).	5
	13b	Describe any methods required to prepare the data for presentation or synthesis, such as handling of missing summary statistics, or data conversions.	Not applicable
	13c	Describe any methods used to tabulate or visually display results of individual studies and syntheses.	Tables 2-3
	13d	Describe any methods used to synthesize results and provide a rationale for the choice(s). If meta-analysis was performed, describe the model(s), method(s) to identify the presence and extent of statistical heterogeneity, and software package(s) used.	9-10
	13e	Describe any methods used to explore possible causes of heterogeneity among study results (e.g. subgroup analysis, meta-regression).	11
	13f	Describe any sensitivity analyses conducted to assess robustness of the synthesized results.	Not applicable
Reporting bias assessment	14	Describe any methods used to assess risk of bias due to missing results in a synthesis (arising from reporting biases).	6
Certainty assessment	15	Describe any methods used to assess certainty (or confidence) in the body of evidence for an outcome.	Not reported
**RESULTS**			
Study selection	16a	Describe the results of the search and selection process, from the number of records identified in the search to the number of studies included in the review, ideally using a flow diagram.	7; Figure 1
	16b	Cite studies that might appear to meet the inclusion criteria, but which were excluded, and explain why they were excluded.	Supplementary Table 5
Study characteristics	17	Cite each included study and present its characteristics.	7; Table 2
Risk of bias in studies	18	Present assessments of risk of bias for each included study.	8; Supplementary

Section and Topic	Item #	Checklist item	Location where item is reported
			Tables 3-4
Results of individual studies	19	For all outcomes, present, for each study: (a) summary statistics for each group (where appropriate) and (b) an effect estimate and its precision (e.g. confidence/credible interval), ideally using structured tables or plots.	Table 3
Results of syntheses	20a	For each synthesis, briefly summarise the characteristics and risk of bias among contributing studies.	10
	20b	Present results of all statistical syntheses conducted. If meta-analysis was done, present for each the summary estimate and its precision (e.g. confidence/credible interval) and measures of statistical heterogeneity. If comparing groups, describe the direction of the effect.	10-11
	20c	Present results of all investigations of possible causes of heterogeneity among study results.	11
	20d	Present results of all sensitivity analyses conducted to assess the robustness of the synthesized results.	Not applicable
Reporting biases	21	Present assessments of risk of bias due to missing results (arising from reporting biases) for each synthesis assessed.	Not assessed
Certainty of evidence	22	Present assessments of certainty (or confidence) in the body of evidence for each outcome assessed.	Not assessed
**DISCUSSION**			
Discussion	23a	Provide a general interpretation of the results in the context of other evidence.	12-13
	23b	Discuss any limitations of the evidence included in the review.	13
	23c	Discuss any limitations of the review processes used.	13
	23d	Discuss implications of the results for practice, policy, and future research.	14
**OTHER INFORMATION**			
Registration and protocol	24a	Provide registration information for the review, including register name and registration number, or state that the review was not registered.	5
	24b	Indicate where the review protocol can be accessed, or state that a protocol was not prepared.	5
	24c	Describe and explain any amendments to information provided at registration or in the protocol.	Not applicable
Support	25	Describe sources of financial or non-financial support for the review, and the role of the funders or sponsors in the review.	16
Competing	26	Declare any competing interests of review authors.	16

Section and Topic	Item #	Checklist item	Location where item is reported
interests			
Availability of data, code and other materials	27	Report which of the following are publicly available and where they can be found: template data collection forms; data extracted from included studies; data used for all analyses; analytic code; any other materials used in the review.	Not reported

Table S2. Search strategies used for each database and number of records retrieved.

Database	Search Strategy	Number of Records Retrieved
PubMed (MEDLINE)	(("oral squamous cell carcinoma"[Title/Abstract] OR "OSCC"[Title/Abstract] OR "squamous cell carcinoma of head and neck"[MeSH Terms]) AND ("saliva"[Title/Abstract] OR "salivary"[Title/Abstract]) AND ("biomarker"[Title/Abstract] OR "biomarkers"[Title/Abstract] OR "biological marker"[Title/Abstract]) AND ("prognosis"[Title/Abstract] OR "predictive value"[Title/Abstract] OR "survival"[Title/Abstract]))	n = 171
Scopus	TITLE-ABS(("oral squamous cell carcinoma" OR "OSCC") AND ("saliva" OR "salivary") AND ("biomarker" OR "biomarkers" OR "biological marker") AND ("prognosis" OR "predictive value" OR "survival"))	n = 163
Web of Science (WoS)	TS=("oral squamous cell carcinoma" OR "OSCC") AND TS=("saliva" OR "salivary") AND TS=("biomarker" OR "biomarkers" OR "biological marker") AND TS=("prognosis" OR "predictive value" OR "survival")	n = 46
Embase	('oral squamous cell carcinoma'/exp OR OSCC:ti,ab) AND (saliva:ti,ab OR 'saliva'/exp) AND (biomarker:ti,ab OR 'biological marker'/exp) AND (prognosis:ti,ab OR 'prognosis'/exp)	n = 190
Cochrane Library	"oral squamous cell carcinoma" AND saliva AND biomarker AND prognosis	n = 2

Table S3. Criteria for judging risk of bias according to the QUIPS tool [2].

Domain	Goal	Key Criteria for Assessment
1. Study Participation	Assess selection bias and whether the study sample represents the target population.	Description of source population, recruitment method, inclusion/exclusion criteria, baseline characteristics.
2. Study Attrition	Evaluate attrition bias due to loss to follow-up.	Proportion available for analysis, reasons for drop-out, comparison of completers vs. non-completers.
3. Prognostic Factor Measurement	Assess bias in the measurement of the prognostic factor (PF).	Clear PF definition, validity/reliability of measurement, method consistency across participants, handling of missing data.
4. Outcome Measurement	Assess bias in outcome measurement.	Clear outcome definition, use of valid/reliable methods, blinding, consistency across participants.
5. Study Confounding	Assess bias due to confounding variables.	Measurement of key confounders, methods to account for confounding in design and analysis.
6. Statistical Analysis and Reporting	Evaluate the appropriateness of the statistical approach.	Transparent model building, suitable statistical methods, absence of selective reporting.

Table S4. Risk of bias analysis of prognostic studies using the QUIPS tool.

Domains	Prompting Items for Consideration	First Author et al., Year														
		Aziz et al., 2015 [23]	Bu et al., 2015 [24]	Wink et al., 2015 [25]	Malhorta et al., 2016 [26]	Pathiyil et al., 2015 [27]	Ko et al., 2018 [28]	Wang et al., 2018 [29]	Zhong et al., 2019 [30]	Romani et al., 2021 [31]	Ishikawa et al., 2022 [32]	Patel et al., 2022 [33]	Shabbir et al., 2022 [34]	Patel et al., 2023 [35]	Premkumar et al., 2023 [36]	Hema Shree et al., 2025 [37]
**Study Participation**	Adequate participation in the study by eligible persons	Partial	Yes	Yes	Yes	Yes	Yes	Yes	Yes	Yes	Yes	Yes	Yes	Yes	Yes	Partial
	Description of the source population or population of interest	Yes	Yes	Yes	Yes	Yes	Yes	Yes	Yes	Yes	Yes	Yes	Yes	Yes	Yes	Yes
	Description of the baseline study sample	Partial	Yes	Yes	Yes	Yes	Yes	Yes	Yes	Yes	Yes	Yes	Yes	Yes	Yes	Yes
	Adequate description of the sampling frame and recruitment	Partial	Yes	Yes	Yes	Yes	Yes	Yes	Yes	Yes	Partial	Yes	Yes	Yes	Yes	No

	Adequate description of the period and place of recruitment	Partial	Yes	Yes	Yes	Yes	Yes	Yes	Yes	Yes	Yes	Yes	Yes	Yes	Yes	Partial
	Adequate description of inclusion and exclusion criteria	Yes	Yes	Yes	Yes	Yes	Yes	Yes	Yes	Yes	Yes	Yes	Yes	Yes	Yes	Partial
	Overall Risk of Bias	*Moderate*	*Low*	*Low*	*Low*	*Low*	*Low*	*Low*	*Low*	*Low*	*Low*	*Low*	*Low*	*Low*	*Low*	*Moderate*
**Study Attrition**	Adequate response rate for study participants	Partial	Yes	Yes	Partial	Yes	Partial	Yes	Yes	Yes	Yes	Partial	Yes	Partial	Yes	Unsure
	Description of attempts to collect information on participants who dropped out	Unsure	Partial	Partial	Unsure	Partial	Unsure	Partial	Partial	Partial	Unsure	Unsure	Partial	Unsure	Partial	No
	Reasons for loss to followup	Unsure	Unsure	Partial	Unsure	Partial	Unsure	Partial	Partial	Partial	Partial	Unsure	Partial	Unsure	Partial	No

	are provided															
	Adequate description of participants lost to followup	Partial	Unsure	Partial	Unsure	Partial	Unsure	Partial	Partial	Partial	Partial	Unsure	Partial	Unsure	Partial	No
	There are no important differences between participants who completed the study and those who did not	Partial	Partial	Unsure	Unsure	Unsure	Unsure	Unsure	Unsure	Unsure	Yes	Unsure	Unsure	Unsure	Unsure	Unsure
	Overall Risk of Bias	*Moderate*	*Moderate*	*Moderate*	*Moderate*	*Moderate*	*Moderate*	*Moderate*	*Moderate*	*Moderate*	*Low*	*Moderate*	*Moderate*	*Moderate*	*Moderate*	*High*
**Prognostic Factor Measurement**	A clear definition or description of the PF is provided	Yes	Yes	Yes	Yes	Yes	Yes	Yes	Yes	Yes	Yes	Yes	Yes	Yes	Yes	Yes
	Method of PF measurement is adequately valid	Partial	Yes	Yes	Yes	Yes	Yes	Yes	Yes	Yes	Yes	Yes	Yes	Yes	Yes	Yes

	and reliable															
	Continuous variables are reported or appropriate cut points are used	Yes	Yes	Yes	Yes	Yes	Yes	Yes	Yes	Yes	Yes	Yes	Yes	Yes	Yes	Yes
	The method and setting of measurement of PF is the same for all study participants	Yes	Yes	Yes	Yes	Yes	Yes	Yes	Yes	Yes	Yes	Yes	Yes	Yes	Yes	Yes
	Adequate proportion of the study sample has complete data for the PF	Yes	Yes	Yes	Yes	Yes	Yes	Yes	Yes	Yes	Yes	Yes	Yes	Yes	Yes	Yes
	Appropriate methods of imputatio	Unsure	Unsure	Unsure	Unsure	Unsure	Unsure	Unsure	Unsure	Unsure	Unsure	Unsure	Unsure	Unsure	Unsure	Unsure

	n are used for missing PF data.															
	Overall Risk of Bias	*Moderate*	*Low*	*Low*	*Low*	*Low*	*Low*	*Low*	*Low*	*Low*	*Low*	*Low*	*Low*	*Low*	*Low*	*Low*
**Outcome Measurement**	A clear definition of the outcome is provided	Yes	Yes	Yes	Yes	Yes	Yes	Yes	Yes	Yes	Yes	Yes	Yes	Yes	Yes	Yes
	Method of outcome measurement used is adequately valid and reliable	Yes	Yes	Yes	Yes	Yes	Yes	Yes	Yes	Yes	Yes	Yes	Yes	Yes	Yes	Partial
	The method and setting of outcome measurement is the same for all study participants	Yes	Yes	Yes	Yes	Yes	Yes	Yes	Yes	Yes	Yes	Yes	Yes	Yes	Yes	Partial
	Overall Risk of Bias	*Low*	*Low*	*Low*	*Low*	*Low*	*Low*	*Low*	*Low*	*Low*	*Low*	*Low*	*Low*	*Low*	*Low*	*Moderate*

**Study Confounding**	All important confounders are measured	Partial	Yes	Partial	Partial	Partial	Partial	Partial	Partial	Partial	Partial	Partial	Partial	Partial	Partial	No
	Clear definitions of the important confounders measured are provided	Partial	Yes	Partial	Yes	Partial	Yes	Partial	Partial	Partial	Yes	Yes	Partial	Yes	Partial	No
	Measurement of all important confounders is adequately valid and reliable	Partial	Yes	Partial	Partial	Partial	Partial	Partial	Partial	Partial	Yes	Partial	Partial	Partial	Partial	Unsure
	The method and setting of confounding measurement are the same for all study participants	Partial	Yes	Partial	Yes	Partial	Yes	Partial	Partial	Partial	Yes	Yes	Partial	Yes	Partial	Unsure

	Appropriate methods are used if imputation is used for missing confounder data	Unsure	Unsure	Unsure	Unsure	Unsure	Unsure	Unsure	Unsure	Unsure	Unsure	Unsure	Unsure	Unsure	Unsure	Unsure
	Important potential confounders are accounted for in the study design	No	Yes	Partial	Yes	Partial	Yes	Partial	Partial	Partial	Partial	Yes	Partial	Yes	Partial	No
	Important potential confounders are accounted for in the analysis	No	Yes	Partial	Yes	Partial	Yes	Partial	Partial	Partial	Yes	Yes	Partial	Yes	Partial	No
	Overall Risk of Bias	*High*	*Low*	*Moderate*	*Moderate*	*Moderate*	*Moderate*	*Moderate*	*Moderate*	*Moderate*	*Moderate*	*Moderate*	*Moderate*	*Moderate*	*Moderate*	*High*
**Statistical Analysis and Reporting**	Sufficient presentation of data to assess the adequacy of the	Yes	Yes	Partial	Yes	Yes	Yes	Partial	Partial	Partial	Yes	Yes	Partial	Yes	Yes	Yes

	analytic strategy															
	Strategy for model building is appropriate and is based on a conceptual framework or model	Partial	Partial	Yes	Yes	Partial	Yes	Yes	Yes	Yes	Yes	Yes	Yes	Yes	Partial	No
	The selected statistical model is adequate for the design of the study	Yes	Yes	Yes	Yes	Yes	Yes	Yes	Yes	Yes	Yes	Yes	Yes	Yes	Yes	Yes
	There is no selective reporting of results	Yes	Yes	Yes	Yes	Yes	Yes	Yes	Yes	Yes	Yes	Yes	Yes	Yes	Yes	Yes
	Overall Risk of Bias	*Low*	*Low*	*Low*	*Low*	*Low*	*Low*	*Low*	*Low*	*Low*	*Low*	*Low*	*Low*	*Low*	*Low*	*Moderate*

Table S5. Summary table of studies excluded in this systematic review.

Excluded Studies	Exclusion Reasons
Brundha et al., 2023 [3]	Prognostic value not investigated
Gai et al., 2018 [4]	Prognostic value not investigated
Gallo et al., 2016 [5]	Prognostic value not investigated
Goncalves et al., 2015 [6]	Prognostic value not investigated
Honarmand et al., 2016 [7]	Prognostic value not investigated
Ishikawa et al., 2021 [8]	Did not met the eligibility criteria
Javaraiah et al., 2020 [9]	Prognostic value not investigated
Jayarajkumar et al., 2023 [10]	Prognostic value not investigated
Kravets et al., 2023 [11]	Tumor sample
Kumari et al., 2021 [12]	Did not met the eligibility criteria
Mohamed et al., 2021 [13]	Prognostic value not investigated
Nandakumar et al., 2020 [14]	Prognostic value not investigated
Oka et al., 2016 [15]	Tumor sample
Sasahira et al., 2017 [16]	Tumor sample
Sawant et al., 2016 [17]	Tumor sample
Scholtz et al., 2022 [18]	Prognostic value not investigated
Tavakoli et al., 2024	Prognostic value not investigated

[19]	
Ueda et al., 2021 [20]	Prognostic value not investigated
Vimal et al., 2023 [21]	Prognostic value not investigated
Wang et al., 2020 [22]	Prognostic value not investigated

References

1. Page MJ, McKenzie JE, Bossuyt PM, Boutron I, Hoffmann TC, Mulrow CD, Shamseer L, Tetzlaff JM, Akl EA, Brennan SE, Chou R, Glanville J, Grimshaw JM, Hróbjartsson A, Lalu MM, Li T, Loder EW, Mayo-Wilson E, McDonald S, McGuinness LA, Stewart LA, Thomas J, Tricco AC, Welch VA, Whiting P, Moher D. The PRISMA 2020 statement: an updated guideline for reporting systematic reviews. BMJ 2021; 372: n71.
2. Hayden JA, van Der Windt DA, Cartwright JL, Côté P, Bombardier C. Assessing bias in studies of prognostic factors. Ann Intern Med. 2013;158(4):280.
3. Brundha MP, Raveendran SR, & Rajeshkar N. Salivary tumour necrosis factor-alpha and receptor for advanced glycation end products as prognostic and predictive markers for recurrence in oral squamous cell carcinoma - a pilot study. European Journal of Clinical and Experimental Medicine 2023; 21(1), 36-43. https://doi.org/10.15584/ejcem.2023.1.5.
4. Gai C, Camussi F, Broccoletti R, Gambino A, Cabras M, Molinaro L, Carossa S, Camussi G, & Arduino PG. Salivary extracellular vesicle-associated miRNAs as potential biomarkers in oral squamous cell carcinoma. BMC Cancer 2018; 18(1), 439. https://doi.org/10.1186/s12885-018-4364-z.
5. Gallo C, Ciavarella D, Santarelli A, Ranieri E, Colella G, Lo Muzio L, & Lo Russo L. Potential Salivary Proteomic Markers of Oral Squamous Cell Carcinoma. Cancer Genomics Proteomics 2016; 13(1), 55-61. https://pubmed.ncbi.nlm.nih.gov/26708599.
6. Gonçalves AS, Arantes DA, Bernardes VF, Jaeger F, Silva JM, Silva TA, Aguiar MC, & Batista AC. Immunosuppressive mediators of oral squamous cell carcinoma in tumour samples and saliva. Human Immunology 2015; 76(1), 52-58. https://doi.org/10.1016/j.humimm.2014.11.002.
7. Honarmand MH, Farhad-Mollashahi L, Nakhaee A, & Nehi M. Salivary Levels of ErbB2 and CEA in Oral Squamous Cell Carcinoma Patients. Asian Pacific Journal of Cancer Prevention 2016; 17(S3), 77-80. https://doi.org/10.7314/apjcp.2016.17.s3.77.
8. Ishikawa S, Ishizawa K, Tanaka A, Kimura H, Kitabatake K, Sugano A, Edamatsu K, Ueda S, & Iino M. Identification of Salivary Proteomic Biomarkers for Oral Cancer Screening. In Vivo 2021; 35(1), 541-547. https://doi.org/10.21873/invivo.12289.
9. Javaraiah RK, David CM, Namitha J, & Tiwari R, Benakanal P. Evaluation of Salivary Lactate Dehydrogenase as a Prognostic Biomarker in Tobacco Users with and without Potentially Malignant Disorders of the Oral Cavity. South Asian Journal of Cancer 2020; 9(2), 93-98. https://doi.org/10.1055/s-0040-1721174.
10. Jayarajkumar S, Ramamoorthi R, Muniapillai S, Gopalakrishnan S, & Jayaseelan VP. Assessment of salivary levels of ErbB2 in oral squamous cell carcinoma. Journal of Oral and Maxillofacial Pathology 2023; 27(4), 777. https://doi.org/10.4103/jomfp.jomfp_114_23.

11. Kravets O, Burtyn O, Borikun T, & Rossylna O. THE STUDY OF PROGNOSTIC VALUE OF microRNAs (miR-10b AND -155) AND CDKN2A/P16INK4A IN ORAL SQUAMOUS CELL CARCINOMA. Experimental Oncology 2023; 45(2), 187-194. https://doi.org/10.15407/exp-oncology.2023.02.187.
12. Kumari P, Syed SA, Wahid M, Qureshi MA, & Kumar R. Expression of miR-31 in saliva-liquid biopsy in patients with oral squamous cell carcinoma. Journal of Taibah University Medical Sciences 2021; 16(5), 733-739. https://doi.org/10.1016/j.jtumed.2021.03.007.
13. Mohamed N, Litlekalsøy J, Ahmed IA, Martinsen EMH, Furriol J, Javier-Lopez R, Elsheikh M, Gaafar NM, Morgado L, Mundra S, Johannessen AC, Osman TA, Nginamau ES, Suleiman A, & Costea DE. Analysis of salivary mycobiome in a cohort of oral squamous cell carcinoma patients from Sudan identifies higher salivary carriage of Malassezia as an independent and favorable predictor of overall survival. Frontiers in Cellular and Infection Microbiology 2021; 11. https://doi.org/10.3389/fcimb.2021.673465.
14. Nandakumar A, Nataraj P, James A, Krishnan R, & KM M. Estimation of Salivary 8-Hydroxydeoxyguanosine (8-OHdG) as a Potential Biomarker in Assessing Progression towards Malignancy: A Case-Control Study. Asian Pacific Journal of Cancer Prevention 2020; 21(8), 2325-2329. https://doi.org/10.31557/APJCP.2020.21.8.2325.
15. Oka R, Nakashiro K, Goda H, Iwamoto K, Tokuzen N, & Hamakawa H. Annexin A8 is a novel molecular marker for detecting lymph node metastasis in oral squamous cell carcinoma. Oncotarget 2016; 7(4), 4882-4889. https://doi.org/10.18632/oncotarget.6639.
16. Sasahira T, Kurihara M, Nishiguchi Y, Nakashima C, Kirita T, & Kuniyasu H. Pancreatic adenocarcinoma up-regulated factor has oncogenic functions in oral squamous cell carcinoma. Histopathology 2017; 70(4), 539-548. https://doi.org/10.1111/his.13097.
17. Sawant S, Gokulan R, Dongre H, Vaidya M, Chaukar D, Prabhash K, Ingle A, Joshi S, Dange P, Joshi S, Singh AK, Makani V, Sharma S, Jeyaram A, Kane S, & D'Cruz A. Prognostic role of Oct4, CD44 and c-Myc in radio-chemo-resistant oral cancer patients and their tumourigenic potential in immunodeficient mice. Clinical Oral Investigations 2016; 20(1), 43-56. https://doi.org/10.1007/s00784-015-1476-6.
18. Scholtz B, Horváth J, Tar I, Kiss C, & Márton IJ. Salivary miR-31-5p, miR-345-3p, and miR-424-3p Are Reliable Biomarkers in Patients with Oral Squamous Cell Carcinoma. Pathogens 2022; 11(2), 229. https://doi.org/10.3390/pathogens11020229.
19. Tavakoli F, Ghavimi MA, Fakhrzadeh V, Abdolzadeh D, Afshari A, & Eslami H. Evaluation of salivary transferrin in patients with oral squamous cell carcinoma. Clinical and Experimental Dental Research 2024; 10(1), e809. https://doi.org/10.1002/cre2.809.
20. Ueda S, Goto M, Hashimoto K, Imazawa M, Takahashi M, Oh-Iwa I, Shimozato K, & Nagao T, Nomoto S. Salivary CPLANE1 Levels as a Biomarker of Oral Squamous Cell Carcinoma. Anticancer Research 2021; 41(2), 765-772. https://doi.org/10.21873/anticanres.14828.
21. Vimal J, George NA, Kumar RR, Kattoor J, & Kannan S. Identification of salivary metabolic biomarker signatures for oral tongue squamous cell carcinoma. Archives of Oral Biology 2023; 155, 105780. https://doi.org/10.1016/j.archoralbio.2023.105780.

22. Wang K, Shen Y, Xu J, Li Z, Liu Y, Yu C, Peng L, Zheng J, & Zeng Y. Evaluation of synuclein-γ levels by novel monoclonal antibody in saliva and cancer tissues from oral squamous cell carcinoma patients. Neoplasma 2020; 67(3), 707-713. https://doi.org/10.4149/neo_2020_190619n523.
23. Aziz S, Ahmed SS, Ali A, Khan FA, Zulfiqar G, Iqbal J, Khan AA, Shoaib M. Salivary Immunosuppressive Cytokines IL-10 and IL-13 Are Significantly Elevated in Oral Squamous Cell Carcinoma Patients. Cancer Investigation 2015; 33(7):318-28. doi: 10.3109/07357907.2015.1041642.
24. Bu J, Bu X, Liu B, Chen F, Chen P. Increased Expression of Tissue/Salivary Transgelin mRNA Predicts Poor Prognosis in Patients with Oral Squamous Cell Carcinoma (OSCC). Medical science monitor: international medical journal of experimental and clinical research 2015; 21:2275-81. doi: 10.12659/MSM.893925.
25. Winck FV, Prado Ribeiro AC, Ramos Domingues R, Ling LY, Riaño-Pachón DM, Rivera C, Brandão TB, Gouvea AF, Santos-Silva AR, Coletta RD, Paes Leme AF. Insights into immune responses in oral cancer through proteomic analysis of saliva and salivary extracellular vesicles. Scientific report 2015; 5:16305. doi: 10.1038/srep16305.
26. Malhotra R, Urs AB, Chakravarti A, Kumar S, Gupta VK, Mahajan B. Correlation of Cyfra 21-1 levels in saliva and serum with CK19 mRNA expression in oral squamous cell carcinoma. Tumour biology: the journal of international society for oncodevolopmental biology and medicine 2016; 37(7):9263-71. doi: 10.1007/s13277-016-4809-4.
27. Pathiyil V, D'Cruz AM. Salivary lactate dehydrogenase as a prognostic marker in oral squamous cell carcinoma patients following surgical therapy. Journal of experimental and therapeutic & oncology 2017; 11(2):133-137. PMID: 28976136.
28. Ko HH, Peng HH, Cheng SJ, Kuo MY. Increased salivary AKR1B10 level: Association with progression and poor prognosis of oral squamous cell carcinoma. Head&Neck 2018 ;40(12):2642-2647. doi: 10.1002/hed.25370.
29. Wang CI, Yu CJ, Huang Y, Yi JS, Cheng HW, Kao HK, Lao WW, Chang KP. Association of overexpressed karyopherin alpha 2 with poor survival and its contribution to interleukin-1β-induced matrix metalloproteinase expression in oral cancer. Head&Neck 2018; 40(8):1719-1733. doi: 10.1002/hed.25145.
30. Zhong WQ, Ren JG, Xiong XP, Man QW, Zhang W, Gao L, Li C, Liu B, Sun ZJ, Jia J, Zhang WF, Zhao YF, Chen G. Increased salivary microvesicles are associated with the prognosis of patients with oral squamous cell carcinoma. Journal of cellular and molecular medicine 2019; 23(6):4054-4062. doi: 10.1111/jcmm.14291.
31. Romani C, Salviato E, Paderno A, Zanotti L, Ravaggi A, Deganello A, Berretti G, Gualtieri T, Marchini S, D'Incalci M, Mattavelli D, Piazza C, Bossi P, Romualdi C, Nicolai P, Bignotti E. Genome-wide study of salivary miRNAs identifies miR-423-5p as promising diagnostic and prognostic biomarker in oral squamous cell carcinoma. Theranostics 2021; 11(6):2987-2999. doi: 10.7150/thno.45157.
32. Ishikawa S, Sugimoto M, Konta T, Kitabatake K, Ueda S, Edamatsu K, Okuyama N, Yusa K, Iino M. Salivary Metabolomics for Prognosis of Oral Squamous Cell Carcinoma. Front Oncol. 2022 ;11:789248. doi: 10.3389/fonc.2021.789248.

33. Patel A, Patel S, Patel P, Mandlik D, Patel K, Tanavde V. Salivary Exosomal miRNA-1307-5p Predicts Disease Aggressiveness and Poor Prognosis in Oral Squamous Cell Carcinoma Patients. International journal of molecular science 2022; 23(18):10639. doi: 10.3390/ijms231810639.
34. Shabbir A, Waheed H, Ahmed S, Shaikh SS, Farooqui WA. Association of salivary Cathepsin B in different histological grades among patients presenting with oral squamous cell carcinoma. BMC Oral Health 2022; 22(1):63. doi: 10.1186/s12903-022-02052-1.
35. Patel A, Patel P, Mandlik D, Patel K, Malaviya P, Johar K, Swamy KBS, Patel S, Tanavde V. A novel 3-miRNA network regulates tumour progression in oral squamous cell carcinoma. Biomarker research 2023; 11(1):64. doi: 10.1186/s40364-023-00505-5.
36. Premkumar A, Walia C, Roy S. The comparative evaluation of salivary survivin levels between different grades of oral squamous cell carcinoma. Journal of biomedicine and biotechnology research 2023; 7:293-7. doi: 10.4103/bbrj.bbrj_83_23.
37. Hema Shree K, Gayathri R, Veeraraghavan VP, Ramani P, Ramadoss R, Yuwanati M. Gold nanoparticle enhanced TNFα antibody interface using saliva for predicting prognosis in OSCC. Arch Oral Biol. 2025;173:106196. doi: 10.1016/j.archoralbio.2025.106196.
